# Supplementary material for: Genomic Characterization of Three Novel Bartonella Strains in a Rodent and Two Bat Species from Mexico
Source: Microorganisms. 2023 Jan 30;11(2):340. doi: 10.3390/microorganisms11020340 (PMC9962129; doi:10.3390/microorganisms11020340)
Supplement: Supplementary file 1 [file microorganisms-11-00340-s001.zip › microorganisms-2194917-supplementary.pdf]

SUPPLEMENTARY MATERIALS

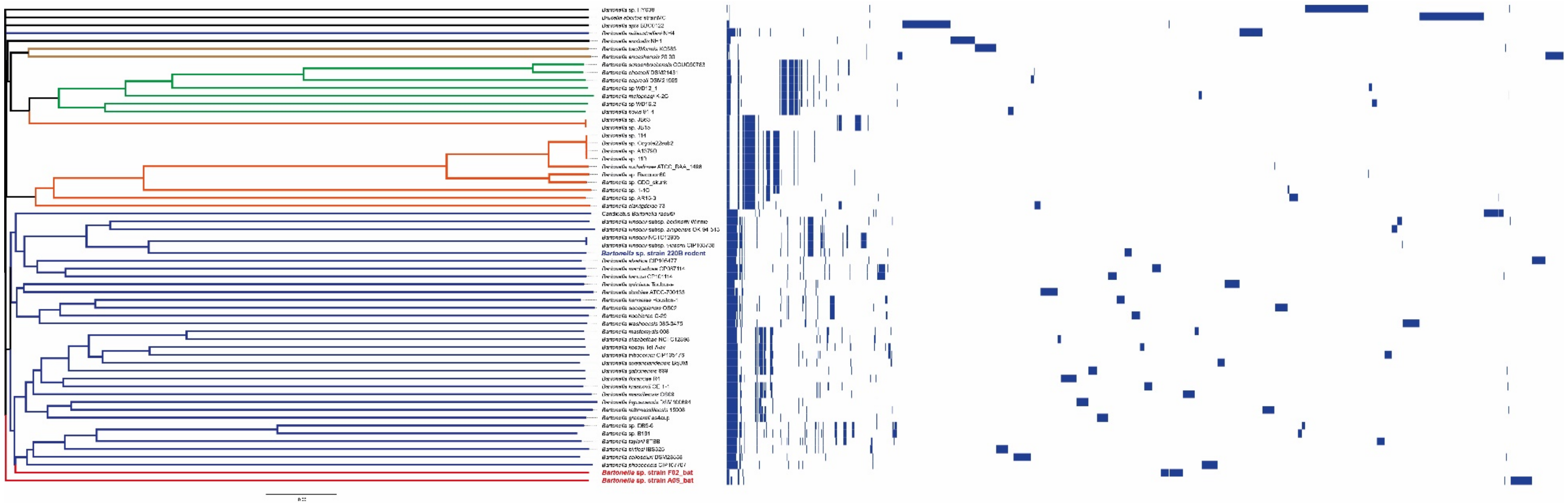

Figure S1. Pan-genome analysis of 60 *Bartonella* genomes: The neighbor-joining phylogenomic tree to the left, colored according to the described lineages: (black: ancestors; brown: lineage 1; green: lineage 2; orange: lineage 3; blue, lineage 4; red: lineage 5). The matrix on the right is the calculated pan-genome for *Bartonella* genomes.
